# Parental Emotional Socialization and Child Mental Health After a Military Parenting Program: A Baseline Target Moderated Mediation Model

**DOI:** 10.1007/s11121-025-01859-y

**Published:** 2025-12-03

**Authors:** Qiyue Cai, Lijun Li, Abigail H. Gewirtz

**Affiliations:** 1 Department of Psychology, REACH Institute, Arizona State University, 900 S McAllister Ave, Room 205, Tempe, AZ USA; 2 Department of Psychology, Pennsylvania State University, University Park, PA USA; 3 Paul Baerwald School of Social Work & Social Welfare, Hebrew University of Jerusalem, Jerusalem, Israel

**Keywords:** Parental emotional socialization, Military families, Prevention, Baseline target moderated mediation

## Abstract

Parental emotional socialization (PES) has been recognized as a critical mechanism in parenting programs to enhance children's well-being, especially following adversity. However, few studies have examined the potential moderating effect of baseline PES levels. This study aimed to examine whether supportive and unsupportive PES can mediate the intervention effects of a parenting program on child adjustment (Aim 1), and whether baseline PES can moderate the effect (Aim 2). This study utilized data from two randomized controlled trials for post-deployed military families (*N* = 335, *Mage* = 8.25, 54% girls). Families were either assigned to in-person intervention condition (*n* = 226) or a treatment-as-usual condition (*n* = 109). Baseline-targeted moderation mediation (BTMM) models were conducted for mothers and fathers separately, with child age, child sex, child minority status, family household income, and deployment length included as covariates. The parenting program had an indirect effect on child internalizing and externalizing problems 1-year post-baseline through reduced maternal unsupportive PES at post-intervention, while no indirect effect was found through supportive PES. Additionally, baseline PES moderated the impact of the ADAPT program on maternal supportive and unsupportive PES post-intervention, such that mothers who reported less supportive PES or more unsupportive PES at baseline benefited more. No intervention effect was found through fathers’ PES. The findings underscore the crucial role of baseline PES in shaping behavioral parenting intervention effectiveness. The study highlights that one size does not fit all and future research and practice should consider the diverse needs and responses of families, emphasizing the delivery of personalized interventions to best meet parents’ needs and maximize support.

## Introduction

From September 11, 2001, to September 2021, millions have served in the US military, with more than half deployed overseas to support post-9/11 operations. Studies have consistently shown the negative impact of deployment and combat exposure on a service member's mental health, including a higher likelihood of developing PTSD (Fulton et al., 2015), increased substance use (Teeters et al., 2017), greater incidence of war-related disability (Clarke et al., 2015), and more frequent suicidal ideation and attempts (Stanley & Larsen, 2019). Additionally, nearly a third of deployed service members were married with children, and about 5.8% were single parents (Department of Defense, 2023). Over two million children experienced a parent's deployment to the post-9/11 conflicts (Department of Defense, 2023). The Military Family Stress Model (Cheng et al., 2024; Gewirtz et al., 2018b) elucidates the impact of deployment stressors on the family via their impact on parental emotional distress, which may further disrupt parenting practices and couple relationships, and ultimately impact children’s emotional and behavioral adjustment. From a developmental perspective, such disruptions are particularly pronounced during middle childhood (Card et al., 2011), highlighting the critical need for effective interventions that address the needs of military families.

From a social interactive learning perspective (SIL; Patterson, 2002), a structured, secure, and emotionally supportive social environment facilitated by effective parenting can significantly enhance family and child well-being. GenerationPMTO (Forgatch et al., 2017) was developed to emphasize key effective parenting skills, including positive involvement, skill encouragement, problem-solving, monitoring, and appropriate discipline. A series of randomized controlled trials have demonstrated that GenerationPMTO interventions have a long-term impact on children's internalizing and externalizing problems by enhancing parenting practices among divorced families, stepfamilies, military families, immigrants, and foster care, as illustrated in a recent meta-analysis (Cai et al., 2022).

While early research on parenting interventions emphasized the importance of reducing coercive discipline and enhancing general behavioral skills (e.g., Forgatch & DeGarmo, 1999), recent studies have increasingly focused on the role of parental emotion socialization (e.g., Havighurst & Kehoe, 2017). Children's abilities to express and regulate emotions are strong protective factors against PTSD as well as internalizing and externalizing problems after trauma exposure (Villalta et al., 2018), and these skills are primarily learned through parent–child interactions. Thus, understanding how parents influence their children’s emotional development is crucial. Parental emotional socialization (PES) refers to the process by which parents teach children how to understand, express, and regulate emotions in a socially appropriate way (Eisenberg, 2020; Eisenberg et al., 1998). PES has been linked with child adjustment, particularly in the context of family adversity or trauma (Hajal & Paley, 2020). For example, a recent review (Lavi et al., 2021) highlighted that parents at risk of child maltreatment often experience and exhibit more negative emotions and are more likely to be emotionally dysregulated. Katz and colleagues (Fainsilber Katz et al., 2016) found maternal emotion socialization can indirectly impact child adjustment through child emotion regulation abilities in families affected by intimate partner violence. Given that deployed military families experience higher levels of adversity and face elevated risks for child maladjustment, research is needed to understand PES in military families.

PES is typically categorized into supportive and unsupportive behaviors. Supportive PES includes encouraging emotional expression, providing comfort to reduce distress, and assisting in problem-solving. Unsupportive PES involves punitive reactions to the child’s emotional expression, expressions of parental distress in response to the child’s emotions, minimizing the seriousness of the child's concerns, or devaluing the child's problems. Previous research suggests that supportive PES plays a crucial role in children’s emotion expression and competency while unsupportive PES is associated with emotion dysregulation and mental health challenges (Hurrell et al., 2015; Lunkenheimer et al., 2007; Nelson et al., 2013; Sanders et al., 2015),

Given its important role in child emotional development, PES has been recognized as a key target and mechanism in behavioral parenting programs aimed at enhancing children's well-being, particularly post-adversity. Parenting interventions with a focus on PES typically involve improving parents’ own emotion awareness and regulation, fostering effective communication about emotions with their children, and assisting their children in regulating their emotions and behaviors in a safe environment (Snyder et al., 2013). A meta-analysis on PES-focused parenting interventions in early childhood reported improvements in a variety of outcomes, including PES, parenting, parental wellbeing, child emotion competency, and child adjustment (England-Mason et al., 2023). Similarly, a systematic review highlighted the promising effect of PES-focused parenting programs on mental health among children and adolescents (Havighurst et al., 2020). Additionally, Van der Put et al. (2018) showed that parenting interventions including enhancing parental emotional and social support are related to better outcomes in child maltreatment treatments, further supporting the significant role of PES in parenting programs, especially in the face of adversity.

Despite the promising benefits of interventions targeting PES, it is important to explore heterogeneous responses to evidence-based parenting interventions since “one size does not fit all” (Shelleby & Shaw, 2014). Some evidence supports the *risk enhancement* hypothesis, indicating that families with higher baseline risks – such as more child adjustment difficulties, parental mental health challenges, lower parenting, and higher socioeconomic risk – benefit more from parenting interventions, as there is more room for improvement after intervention (e.g., Nowak & Heinrichs, 2008; Shelleby & Shaw, 2014; Zhang et al., 2023). Conversely, some studies find support for the *risk buffering* effect, that families with lower baseline risk benefit more (Chesmore et al., 2018; Lundahl et al., 2006), as some high-risk families request more intense resources for change than can be offered in a prevention program. There also are data suggesting that parenting interventions are *universally effective*, showing no moderation effects (e.g., Weeland et al., 2017).Therefore, taking the baseline level of risk into account is helpful in determining the effectiveness of parenting interventions as well as in tailoring programs to meet families’ unique needs and optimize their benefits (Ng & Weisz, 2016). One way to better understand these inconsistencies is to examine the potential moderating role of baseline levels of targeted mediators, or the theorized malleable risk or protective factors. This approach, known as baseline target moderated mediation (BTMM; Howe, 2019; Howe & Leijten, 2023; Tein et al., 2004; Valente et al., 2025), has its unique strengths by integrating both moderation and mediation analyses within a single model, which can clarify the conditional effects of the mediation mechanisms. In other words, BTMM can help us understand whether intervention effects are influenced by pre-intervention status and identify important tailoring variables for future adaptation of parenting interventions.

In sum, existing literature highlights the critical role of PES in child development and its function as a key mechanism in intervention programs aimed at enhancing child adjustment. However, research on PES in military families remains limited, despite the unique stressors and challenges they face post-deployment. Additionally, few studies have examined the role of baseline PES in altering the effectiveness of parenting programs within this population. While evidence-based parenting interventions have been shown to significantly improve family well-being post-deployment (Gewirtz et al., 2018a; N. Zhang et al., 2018, 2020), it remains unclear whether baseline PES moderates the effectiveness of these programs. Without this knowledge, interventions may not reach their full potential.

### The Current Study

**Fig. 1:**
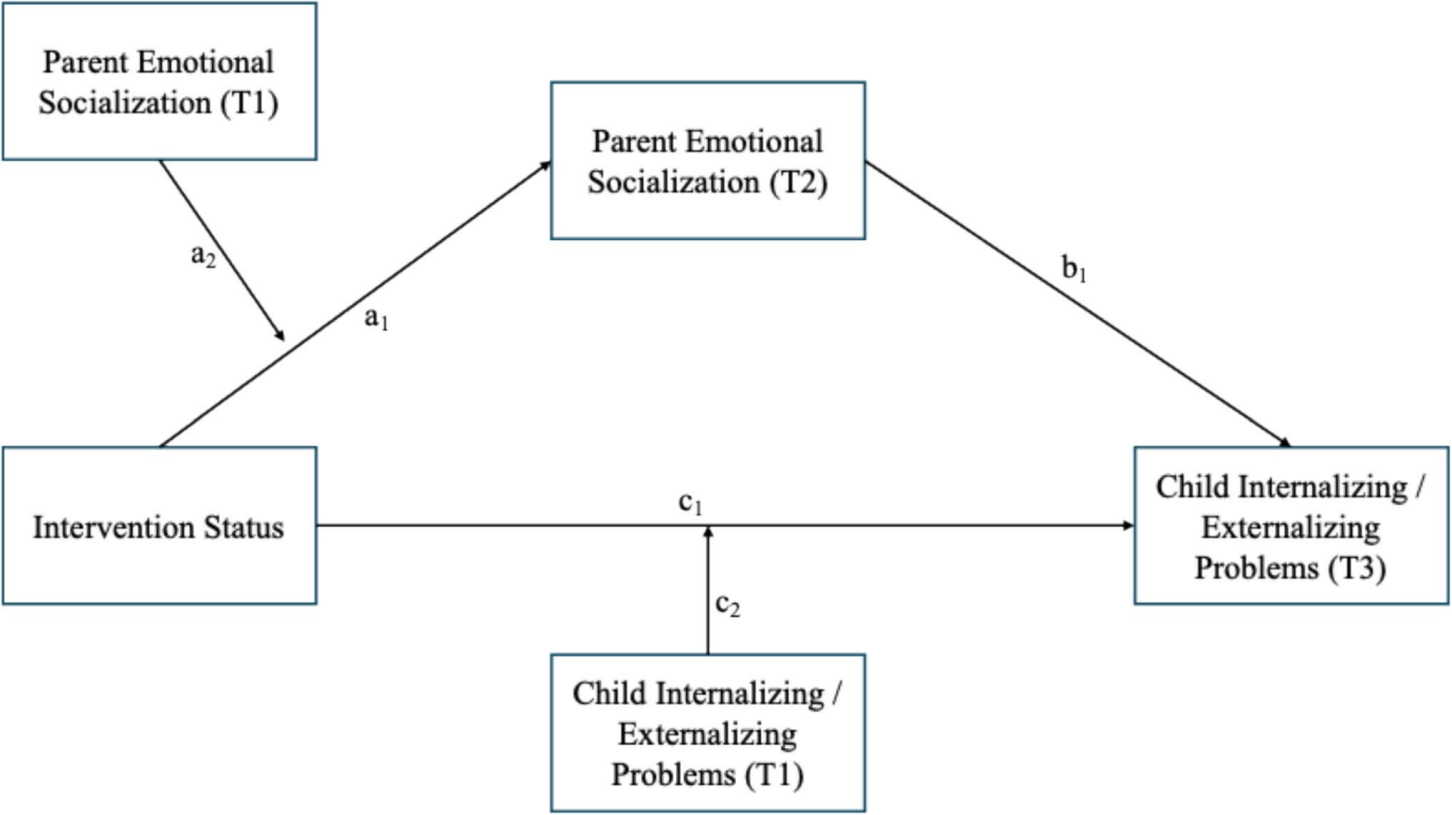
Conceptual model

To fill these gaps, this study aimed to examine whether supportive and unsupportive PES at post-treatment can mediate the relationship between a parenting program and child internalizing and externalizing problems (Aim 1) and whether baseline PES can moderate the relationship between the ADAPT program and emotional socialization at post-treatment (Aim 2). ADAPT is a trauma- and mindfulness-informed adaptation of GenerationPMTO (Gewirtz et al., 2014). The ADAPT program modified GenerationPMTO to add a core component, parental emotion socialization, which included low-dose mindfulness practices, emotion coaching skills, and discussions about specific stressors relevant to military families. We examined data from two randomized controlled trials (RCTs) of the After Deployment, Adaptive Parenting Tools (ADAPT) intervention; the first, known as the ADAPT trial, an effectiveness trial with a no-treatment control condition; the second, known as ADAPT4U, a comparative effectiveness trial of three intervention formats of the program. A conceptual model can be found in Fig. 1.

## Methods

### Participants

This study is a secondary data analysis of two RCTs of a preventive parenting program for post-deployed military families. Eligibility criteria included having at least one parent who had been deployed to the post 9/11 conflicts (i.e., Operation Iraqi Freedom, Operation Enduring Freedom, and Operation New Dawn) and at least one child between the ages of 4 and 12.

The final sample included 335 two-parent military families, including 164 from ADAPT and 62 from ADAPT4U in the intervention condition, and the 109 families in the treatment-as-usual control condition from ADAPT. On average, parents were in their thirties (Mother: *M* = 35.84, *SD* = 5.76; Father: *M* = 37.7 years, *SD* = 6.39). Most parents identified as White (90.7% of mothers and 85.1% of fathers) and married (93.1% of mothers and 91.6% of fathers). Most fathers had been deployed (94.3%) and most mothers (85.1%) were civilians. Median household income was reported to be $51,000 – $80,000. The mean age of the target children was 8.25 years (*SD* = 2.49). More than half of the children were girls (54%) and about 17.3% were racial minorities.

### Procedure

Families were recruited through multiple approaches including posted flyers, advertising on social media, National Guard and Reserve deployment and reintegration events, and word-of-mouth referrals from families. After initial eligibility screening and consent, eligible families completed the baseline assessment (T1). In the original trial (ADAPT; Gewirtz et al., 2018a), families were then randomly assigned to one of the two conditions: in-person group (*n* = 207) or a treatment-as-usual control condition (*n* = 129) which provided web and print parenting resources. The second trial (ADAPT4U; Gewirtz et al., 2024) included 244 families who were randomized into various intervention formats, including in-person groups (*n* = 95), individual telehealth (*n* = 71), and self-directed online (*n* = 78) conditions. To ensure consistency in intervention conditions and to maximize statistical power, we combined the in-person treatment groups from both trials. Only two-parent families were included in the current analysis to better represent family dynamics. The final sample included 335 military families, including the in-person intervention group (*n* = 164 from ADAPT and 62 from ADAPT4U) and the treat-as-usual control group (*n* = 109). Follow-up assessments were conducted at post-treatment (T2; six-month post baseline) and then 12- (T3) and 24-month (T4) follow-up; data in this study are focused on T1-T3.

### Intervention

Families assigned to the in-person treatment group in both ADAPT and ADAPT4U were grouped into 5–10 families who participated in 14 in-person weekly sessions with two or three ADAPT facilitators at community locations (e.g., schools or public libraries) chosen based on geographic proximity to the families. Parenting skills were taught each week, and each new skill built on prior skills. Each session followed the same format, with a review of the home practice exercise, a modeling of the skill to be taught with role play, discussion and other active teaching tools, and provision of the new home practice exercise. Each session included a brief mindfulness exercise, and several sessions focused on supportive emotion coaching skills. All sessions were videotaped to ensure fidelity. In addition to the group sessions, families had access to an online ADAPT course containing video demonstrations of the skills for additional practice. Parents received phone calls from facilitators between sessions to address questions and troubleshoot home practice assignments.

### Measures

#### Intervention Status

The current study adopted an intent-to-treat approach to estimate the intervention effect. Thus, intervention status was dummy coded (−1 = treatment-as-usual control condition; 1 = in-person treatment condition).

#### Parental Emotion Socialization

Parental emotional socialization was measured by The Coping with Children's Negative Emotions Scale (CCNES; Fabes et al., 2002) at T1 and T2. Parents read 12 scenarios in which their children may experience negative emotions (e.g., “*If my child loses some prized possession and reacts with tears*”) and then were asked to report their responses on a 7-point Likert scale (1 = *very unlikely*, 7 = *very likely*). The CCNES consists of six subscales, including emotion-focused reaction (e.g., “*distract my child by talking about happy things*”), problem-focused reaction (e.g., “*help my child think of places he/she hasn't looked yet*”), expressive encouragement (e.g., “*tell him/her it's OK to cry when you feel unhappy*”), minimization reaction (e.g., “*tell my child that he/she is over-reacting*”), punitive reaction (e.g., “*tell him/her that's what happens when you're not careful*”), and distress reaction (e.g., “*get upset with him/her for being so careless and then crying about it*”). Emotion-focused reaction, problem-focused reaction, and expressive encouragement are considered supportive parental emotional coaching, and minimization reaction, punitive reaction, and distress reaction are considered unsupportive parental emotional coaching. The current study used the composite scores of supportive and unsupportive parental emotional coaching. Cronbach’s α for the current sample demonstrated good reliability for supportive and unsupportive PES at T1 and T2 (αs >.75 for mothers and αs >.80 for fathers).

#### Child Internalizing and Externalizing Problems

Child internalizing and externalizing problems were measured by the Behavior Assessment System for Children (BASC-2; Reynolds & Kamphaus, 2006) at T1 and T3. Both parents were asked to report the frequency of children’s behaviors on a 4-point Likert scale (0 = *never*, 3 = *almost always*). The composite score for internalizing problems includes symptoms measured by depression (e.g., “*cries easily*”), anxiety (e.g., “*worries*”), and somatization (e.g., “*complains of pain*”) subscales. The composite score for externalizing problems includes symptoms measured by hyperactivity (e.g., “*acts out of control*”), aggression (e.g., “*bullies others*”), and conduct problems (e.g., “*breaks the rules*”) subscales. Age- and gender- adjusted T-scores (*M* = 50, *SD* = 10) for internalizing and externalizing symptoms were calculated and used in the current analysis. Cronbach’s α for the current sample demonstrated good reliability for internalizing symptoms and externalizing symptoms at T1 and T3 (αs >.70 for mothers and αs >.80 for fathers, other than α =.68 for internalizing problems at T1 for ADAPT4U group).

#### Covariates

To control for potential confounding effects, child age (in years), total lengths of deployment (in months), and household income were included as continuous covariates. Dummy coded child gender (0 = girls, 1 = boys), race (0 = White, 1 = people of color), and marital status (0 = divorced/separated/widowed/never married, 1 = married/domestic partnership) were included as categorical covariates.

### Analytic Plan

First, descriptive analyses were first conducted in SPSS 28.0 (IBM Corp, 2021) to obtain the characteristics of the study sample as well as the variables. Bivariate correlations were computed to explore the associations among variables. One-way ANOVA and chi-square tests were conducted to investigative baseline differences among groups.

Next, baseline targeted moderated mediation models (BTMM) were conducted. BTMM integrates mediation and moderation by estimating indirect effect through the targeted mediator (mediation), while allowing those indirect effects to vary as a function of baseline mediator levels (moderation). In other word, the BTMM approach examines the *how* an intervention works (the hypothesized mechanism of change) and *for whom* the mechanism of change works better within a single model. In this study, supportive or unsupportive PES at T2 was included as a mediator in the association between the intervention status and child internalizing and externalizing problems measured at T3. Supportive or unsupportive PES at T1 was included as a moderator in the path from intervention status to PES at T2. Baseline child internalizing and externalizing problems were also included as moderators in the path from intervention status to child internalizing and externalizing problems measured at T3. Given the correlations between mother and father reports of child adjustment especially for internalizing problems were moderately positive instead of strongly positive (all *rs* <.50), both parents' reports were included as outcomes separately. Due to the low correlation between mother and father reports of PES on both supportive and unsupportive PES subscales (all *rs* <.30), we retained both parents’ reports separately in the analysis. Thus, a total of four models were conducted, each with separate mediators (Mediator 1: supportive PES; mediator 2: unsupportive PES) and outcomes (outcome 1: internalizing problems; outcome 2: externalizing problems).

All models were analyzed in Mplus 8 (Muthen & Muthen, 1998–2017). As Little’s MCAR test (Little, 1988) was not statistically significant (*χ*^*2*^ (397) = 424.06, *p* = 0.17), full information maximum likelihood (FIML) estimation was used to handle missing data. The maximum likelihood robust (MLR) estimator was used in model estimation.

## Results

### Descriptive Results

Sample characteristics of the full sample, and three groups are presented in Table 1. Children in the ADAPT4U intervention group were significantly younger than those in the ADAPT intervention group or the control group. Mothers in the ADAPT4U intervention group were deployed longer than the other two groups. Mothers in the control group reported lower levels of supportive parental emotion socialization than the ADAPT intervention condition at baseline. Mothers reported children in the ADAPT4U intervention group as having lower levels of externalizing problems compared to the control group, while fathers reported children in the ADAPT4U intervention group as having lower levels of externalizing problems compared to the control group and the ADAPT intervention group. No other significant group differences were identified at baseline. The means, standard deviations, and bivariate correlations for all main variables can be found in Table 2.

**Table 1:** *Summary of participant demographic information for the total sample, and from each of the group included.*

	Full Sample	Control^a^	ADAPT Group^b^	ADAPT4U Group^c^	ANOVA/Chi square
Family *N*	335	109	164	62	
Child Age *M (SD)*	8.25 (2.49)	8.67 (2.47)	8.25 (2.54)	7.52 (2.22)	c < a* (*p*=.011)
Girl *n (%)*	54%	56%	55.5%	46.9%	X (334) = 1.62
Child of Color	17.3%	20.2%	15.9%	16.1%	X (334) =.932
Mother Age	35.84 (5.76)	35.49 (6.13)	35.93 (5.67)	36.23 (5.35)	*F* (2, 324) =.355
Father Age	37.7 (6.39)	37.39 (6.81)	37.52 (6.18)	38.72 (6.14)	*F* (2, 324) =.971
Married or Cohabiting *n (%)*	93.7%	94.4%	93.3%	93.7%	X (334) =.150
Mother Deployment Length	2 (5.83)	2.09 (6.24)	1.36 (4.31)	3.55 (8.03)	c < b * (*p* = .039)
Father Deployment Length	18.58 (11.45)	18.06 (11.56)	19.60 (11.27)	16.80 (11.63)	*F* (2, 329) = 1.501
Household Income (*Med*)	$51,000—80,000	$51,000—80,000	$51,000—80,000	$51,000—80,000	
Supportive PES Mom T1	5.52 (0.64)	5.42 (0.61)	5.64 (0.62)	5.41 (0.71)	a < b* (*p* = .020)
Supportive PES Dad T1	5.08 (0.81)	5.11 (0.77)	5.1 (0.79)	4.98 (0.91)	*F* (2, 323) =.615
Unsupportive PES Mom T1	2.58 (0.59)	2.53 (0.62)	2.59 (0.57)	2.63 (0.58)	*F* (2, 327) =.657
Unsupportive PES Dad T1	2.88 (0.75)	2.88 (0.75)	2.91 (0.74)	2.78 (0.81)	*F* (2, 325) =.760
Externalizing Dad T1	57.1 (14.43)	58.89 (14.05)	58.08 (15.68)	51.71 (9.98)	c < a** (*p* = .006),c < b** (*p* = .009)
Externalizing Mom T1	56.39 (14.04)	58.4 (14.55)	56.48 (14.55)	52.72 (11.00)	c < a* (*p* = .036)
Internalizing Dad T1	51.07 (9.96)	52.57 (10.37)	50.64 (9.97)	49.67 (9.01)	*F* (2, 316) = 1.908
Internalizing Mom T1	52.09 (11.77)	52.49 (12.08)	51.48 (11.33)	53.03 (12.45)	*F* (2, 318) =.460

*Note*. Post-hot comparison with Bonferroni correction was reported for those with significant group differences

*p <.05. **p <.01. ***p <.001

**Table 2:** Mean scores, standard deviations, and bivariate correlations among all main variables

	1	2	3	4	5	6	7	8	9	10	11	12	13	14	15	16
1. Supportive PES Mom T1	1.00															
2. Supportive PES Dad T1	.18**	1.00														
3. Unsupportive PES Mom T1	‒.27***	‒.12*	1.00													
4. Unsupportive PES Dad T1	‒.04	‒.29***	.19***	1.00												
5. Supportive PES Mom T2	.62**	.10	‒.14*	‒.02	1.00											
6. Supportive PES Dad T2	.15*	.56***	‒.21**	‒.29***	.12	1.00										
7. Unsupportive PES Mom T2	‒.22**	‒.09	.65***	.11	‒.29***	‒.15*	1.00									
8. Unsupportive PES Dad T2	‒.08	‒.29***	.13	.57***	‒.11	‒.53***	.11	1.00								
9. Externalizing Dad T1	.04	.01	.02	.15**	.07	‒.10	‒.06	.03	1.00							
10. Externalizing Mom T1	.01	.06	.15**	.05	.08	‒.04	.04	‒.06	.79***	1.00						
11. Internalizing Dad T1	-.05	-.09	.12*	.17**	‒.08	‒.13	.08	.22**	.19***	.06	1.00					
12. Internalizing Mom T1	‒.04	‒.01	.19***	‒.03	‒.09	.09	.15*	‒.01	‒.01	.18**	.48***	1.00				
13. Externalizing Dad T3	.00	‒.10	‒.06	.19**	‒.01	‒.10	‒.03	.11	.57***	.50***	.16*	.08	1.00			
14. Externalizing Mom T3	‒.07	.00	.12	.16*	.01	‒.15	.14*	.06	.46***	.62***	.06	.26***	.70***	1.00		
15. Internalizing Dad T3	‒.01	-.03	.03	.11	.00	‒.05	.00	.18*	.23***	.17**	.65***	.36***	.46***	.25***	1.00	
16. Internalizing Mom T3	‒.08	.00	.21***	.03	‒.11	‒.04	.25**	.10	.07	.20**	.36***	.67***	.17**	.43***	.49***	1.00
Mean	5.52	5.08	2.58	2.88	5.52	5.08	2.45	2.79	57.10	56.39	51.07	52.09	52.66	53.28	50.57	52.03
SD	0.64	0.81	0.59	0.75	0.71	0.81	0.60	0.70	14.43	14.04	9.96	11.77	10.87	11.44	10.50	11.29
N	330	326	330	326	228	199	228	199	320	319	319	321	250	261	250	262

*p <.05. **p <.01. ***p <.001

### Baseline Moderated Mediation

All results can be found in Table 3.

**Table 3:** Moderated mediation models assessing ADAPT on children’s internalizing and externalizing behaviors via parental supportive and unsupportive PES. All coefficients are unstandardized

	Parent	Mediator	Outcome	a_1_ (SE)	b_1_ (SE)	c_1_ (SE)	a_2_ (SE)	c_2_ (SE)	Indirect
M1	Mother	Supportive PES	Internalizing	0.06 (.04)	‒0.48 (.76)	‒1.30 (.45)**	‒0.12 (.06)*	‒0.50 (.04)	‒0.03 (.05)
	Father	Supportive PES	Internalizing	0.02 (.05)	0.81 (.73)	‒1.30 (.45)**	0.04 (.09)	−0.01 (.07)	0.02 (.04)
M2	Mother	Supportive PES	Externalizing	0.06 (.04)	‒0.48 (.64)	‒0.47 (.53)	‒0.11 (.06)*	0.004 (.05)	‒0.03 (.05)
	Father	Supportive PES	Externalizing	0.02 (.05)	0.18 (.67)	‒0.47 (.53)	0.04 (.09)	0.01 (.05)	0.004 (.02)
M3	Mother	Unsupportive PES	Internalizing	‒0.13 (.03)***	2.73 (.86)**	‒1.17 (.45)**	‒0.11 (.05)*	‒0.04 (.04)	‒0.35 (.14)*
	Father	Unsupportive PES	Internalizing	‒0.02 (.04)	0.26 (1.00)	‒1.17 (.45)**	‒0.04 (.06)	‒0.02 (.07)	‒0.004 (.02)
M4	Mother	Unsupportive PES	Externalizing	‒0.13 (.03)***	2.40 (.68)***	‒0.38 (.54)	‒0.11 (.05)*	0.01 (.05)	‒0.31 (.13)*
	Father	Unsupportive PES	Externalizing	‒0.02 (.04)	0.38 (.87)	‒0.38 (.54)	‒0.04 (.06)	0.02 (.05)	‒0.01 (.02)

***p <.001; **p <.01; *p <.05; † <.10. M1 – M4: Model 1 – Model 4

#### Supportive PES

##### Child Internalizing Problems

**Fig. 2:**
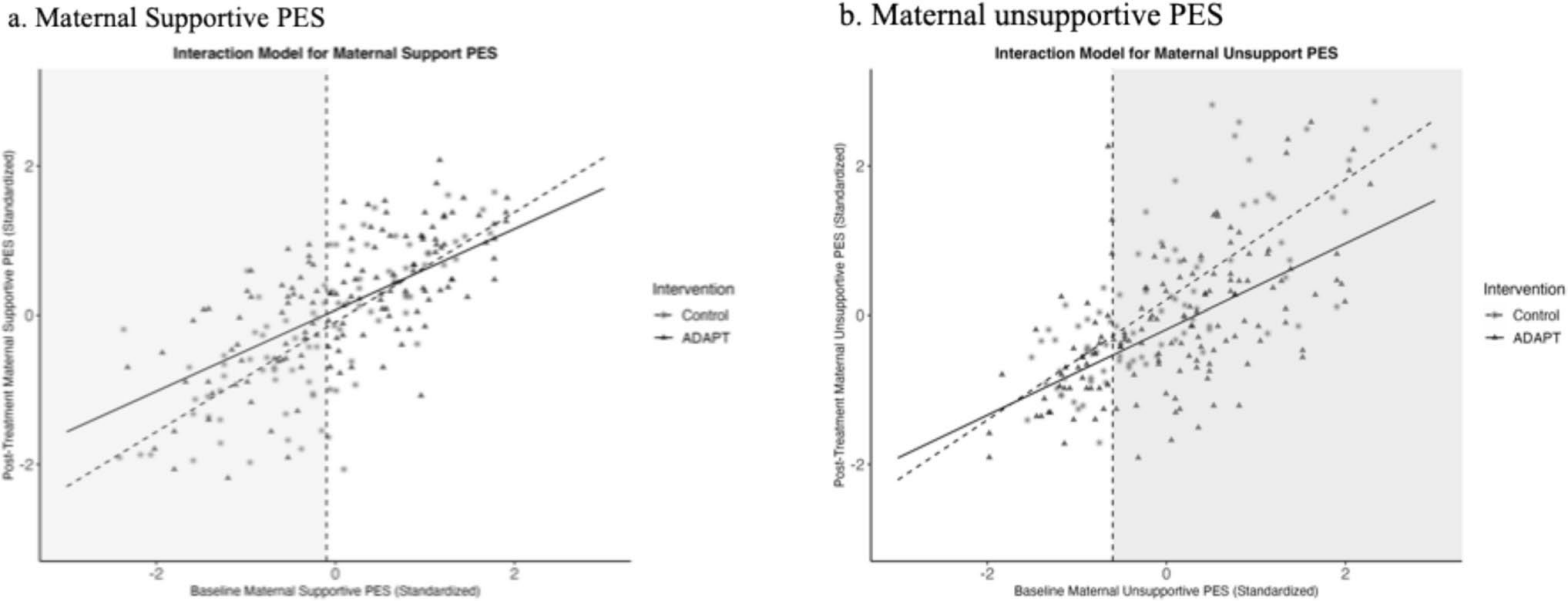
Interaction plot of model depicting interaction between ADAPT and maternal supportive PES (**a**, left) and maternal unsupportive PES (**b**, right), with areas of statistical significance shade

The model showed good model fit: *χ*^*2*^(86) = 116.359, *p* =.016; CFI = 0.929; RMSEA = 0.033, 90% CI = [.015.048]; SRMR =.045. There was a significant direct effect of the intervention on both mother-reported and father-reported child internalizing at T3, and baseline internalizing problem severity did not moderate these direct effects. Although the overall impact of the ADAPT intervention on parents' supportive PES at T2 was not significant, baseline mothers’ supportive PES significantly moderated the path from intervention status to mothers’ supportive PES at T2. As shown in Fig. 2a, mothers with lower baseline supportive PES (below the mean of −0.1 standard deviations) showed significant improvements post-intervention. Baseline fathers’ supportive PES did not moderate the intervention effect on father’s supportive PES at T2.

##### Child Externalizing Problems

The model has acceptable model fit: *χ*^*2*^(86) = 138.758, *p* <.001; CFI = 0.878; RMSEA = 0.044, 90% CI = [.030.057]; SRMR =.054. There was no significant direct effect of intervention on mother-reported or father-reported child externalizing problems measured at T3, nor were these effects moderated by baseline parent-reported child externalizing problems. Similar to the results for child internalizing problems, although the overall impact of the ADAPT intervention on parents' supportive PES at T2 was not significant, baseline mothers’ supportive PES significantly moderated the path from intervention status to mothers’ supportive PES at T2. Since moderation involved the same variable, Fig. 2a is used to illustrate significant improvements in maternal supportive PES at T2 for those with lower baseline supportive PES. Baseline fathers’ supportive PES did not moderate the intervention effect on father’s supportive PES at T2.

#### Unsupportive PES

##### Child Internalizing Problems

The model showed good model fit: *χ*^*2*^(86) = 104.250, *p* =.088; CFI = 0.964; RMSEA = 0.026, 90% CI = [0.042]; SRMR =.044. There was a significant intervention effect on mother-reported child internalizing problems at T3, which was mediated by reduced maternal unsupportive PES at T2. The direct effect was not moderated by baseline problem severity. Importantly, baseline mothers’ unsupportive PES significantly moderated the path from intervention status to mothers’ unsupportive PES at T2. As shown in Fig. 2b, mothers with higher baseline unsupportive PES (above the mean of −0.6 standard deviations) showed significant improvements post-intervention.

A significant direct effect of the intervention on father-reported child internalizing problems at T3 was found. However, this effect was not mediated by fathers’ unsupportive PES at T2. Additionally, no moderation effects were found for baseline severity of internalizing problems or baseline fathers’ unsupportive PES.

##### Child Externalizing Problems

The model showed acceptable model fit: *χ*^*2*^(86) = 138.479, *p* <.001; CFI = 0.897; RMSEA = 0.044, 90% CI = [.030.057]; SRMR =.050. While there was no significant direct intervention effect on mother-reported child externalizing problems at T3, the indirect effect through mothers’ unsupportive PES at T2 was significant. The direct effect was not moderated by baseline problem severity. Baseline mothers’ unsupportive PES significantly moderated the path from intervention status to mothers’ unsupportive PES at T2 (Fig. 2b), with mothers with higher unsupportive PES benefited more at T2.

There was no significant direct or indirect effect of the intervention on father-reported child internalizing problems at T3. Additionally, no moderation effects were found for baseline severity of internalizing problems or baseline fathers’ unsupportive PES.

## Discussion

Utilizing data from two randomized controlled trials, this study employed baseline target moderated mediation models (BTMM) to examine the mediating roles of supportive and unsupportive PES in enhancing child adjustment in a parenting program designed for military families. Our findings partially supported the risk enhancement hypothesis, that mothers with lower baseline supportive and higher baseline unsupportive PES improved more in their PES after the intervention, but only the reductions in unsupportive PES were significantly associated with reduced children’s internalizing and externalizing problems. Baseline children’s problem severity did not moderate the intervention effect. The results highlight the critical role of PES in child adjustment, providing insights for developing and refining personalized interventions tailored to the specific needs of military families.

The findings of this study demonstrated the effectiveness of the ADAPT program in improving maternal PES, but the effect sizes differed based on baseline mediator levels. Consistent with prior findings, our study confirms that parenting interventions can effectively reduce unsupportive PES behaviors, aligning with outcomes reported in systematic reviews and meta-analyses (England-Mason et al., 2023; Havighurst et al., 2020). The ADAPT program puts a strong emphasis on emotion coaching (Snyder et al., 2013), including techniques to improve parents’ awareness and regulation of their own emotions, communication with children about emotions, and the ability to assist children in emotion expression and regulation (Gewirtz et al., 2018a). We found that the ADAPT program is particularly beneficial for mothers who initially show more unsupportive PES, aligning with the risk enhancement hypothesis. The more substantial improvements among mothers with initial struggles with PES are likely because they had more room for growth. In general, given that this was a prevention sample, parents generally showed high supportive and low unsupportive PES at baseline. From a differential susceptibility perspective (Belsky & Pluess, 2009; Van Ijzendoorn et al., 2020), it is also possible that those who struggled more in unsupportive environments also benefit more in supportive contexts, such as intervention. As parents with a history of adversity are more likely to experience difficulties with emotion regulation and emotion socialization (Lavi et al., 2021), future research should investigate whether the techniques used in the ADAPT programs can benefit other prevention and clinical populations. More dismantling studies (e.g., Fortier et al., 2011) are also needed to pinpoint which specific techniques and components of the intervention most effectively change PES, particularly among high-risk populations. Such studies are crucial for determining the appropriate intervention dosage and understanding the mechanisms of change in PES, which can provide important insights into how to tailor interventions to better meet the diverse needs of families, enhancing the effectiveness of parenting interventions across different risk levels.

Mediation analyses indicated that mothers’ unsupportive PES acts as a mediator between the intervention and child internalizing and externalizing problems among military families, consistent with previous research emphasizing the critical role of emotional socialization in child development (Hajal & Paley, 2020). According to the military family stress model (Gewirtz et al., 2018b), deployment and other stressful military transitions can lead to emotional distress among parents, which negatively influences parenting behaviors, couple relationships, and ultimately child well-being. Improvements in PES have the potential to disrupt this cycle. As parents are better able to respond to their own and their child(ren)’s emotions, children benefit from more consistent emotional guidance and support, learning crucial emotion regulation skills via healthy parent–child interactions. Interestingly, findings indicated that reducing unsupportive PES seems to be more important, with supportive PES not significantly correlated to children’s internalizing and externalizing problems in this sample.

From a domain-specific perspective (Grusec & Davidov, 2010), it is likely that while unsupportive PES is related to child adjustment difficulties, supportive PES might be an important resource for children’s adaptive functioning. For example, Nelson et al. (2013) found that supportive (but not unsupportive) PES was related to children’s school and social competency. Lunkenheimer et al. (2007) found an interaction between supportive and unsupportive PES, such that emotion coaching on negative emotions but not positive emotions buffered the impact of emotion dismissing on child outcomes. Future research should further delineate the nuanced differences between supportive and unsupportive PES. A deeper understanding of these distinctions is critical for the development of targeted intervention techniques that more effectively enhance supportive PES and/or reduce unsupportive PES. Such differences can also enhance the selection and tailoring of intervention components that are best suited to particular areas of need within different family contexts.

While this study did not detect immediate intervention effects on paternal PES or indirect effects on child outcomes, another study (N. Zhang et al., 2020) using the same intervention sample revealed improvements over two years in unsupportive PES for both mothers and fathers. This suggests that changes in fathers' PES might require prolonged engagement and practice even after intervention, potentially due to the unique challenges they face post-deployment. For example, another study with this sample (J. Zhang et al., 2023) found that fathers with greater emotion regulation difficulties at baseline exhibited more significant improvements in their observed parenting skills after one year, particularly in responding to children’s emotions. Combined, these findings suggest that fathers facing greater challenges in emotion regulation and emotion socialization may require more time to practice and assimilate the strategies offered by the intervention. Given that the majority of fathers in our study had been deployed overseas and exhibited more struggles with PES compared to mothers, it is hard to disentangle key intervention effects with regard to gender, deployment, trauma exposure, or varying levels of struggles in PES, as it is possible that people with more struggles need more time to practice the techniques acquired from the intervention. Thus, it is important to conduct more longitudinal research to explore how these factors influence parents’ improvements in emotional regulation and emotional socialization, which in turn impacts children’s well-being. Understanding these dynamics is essential, as the complicated, likely bidirectional relationships, will help researchers identify specific areas via which interventions can be tailored to better support these fathers, enhancing familial resilience and well-being.

This study is not without limitations. First, while this paper investigated treatment X baseline mediator and treatment X baseline outcome interactions, the BTMM design has challenges to test multiple moderators simultaneously or three-way interactions (e.g., treatment X baseline mediator X baseline outcome). When interpreting the results, probing conditional indirect effects based on arbitrary values (e.g., ± 1 SD from the mean) instead of a combination of multiple moderators ignores heterogeneity that may exists within subgroups. Future studies should deepen our understanding in differential effectiveness via simulating conditional indirect effects across multiple moderators and incorporating causal mediation frameworks in which mediator-outcome relationship vary as a function of the treatment condition (MacKinnon et al., 2020; Valente et al., 2025). Second, the current study only included parent self-report data; incorporating observational parenting data may lead to different findings, as previous studies have shown differences in self-report parenting and observed parenting (Herbers et al., 2017). Third, we only included participants assigned to the in-person group instead of other intervention components due to considerations related to statistical power, which might overlook potential differences in improvements across different intervention formats (Cotter et al., 2013). While previous research showed different impacts of supportive vs. unsupportive PES on child adaptive vs. maladaptive outcomes (e.g., Nelson et al., 2013), the current study was not able to include child adaptive functioning. Additionally, to better understand family dynamics, we only included two-parent families. The current study also found that PES seems to not correlate between parents, and further research should examine ways to improve PES for both parents. Last but not least, the sample incorporated predominantly White, middle-class, military families which might limit the generalizability of the findings.

Despite these limitations, the study offers important clinical and research implications. Using data from two randomized controlled trials, we applied BTMM to understand how baseline supportive and unsupportive PES might impact intervention response. Our results showed that mothers with less supportive PES and higher unsupportive PES significantly improved their PES after the intervention. By accounting for the moderating role of baseline PES instead of simply including it as a covariate, the results have the potential to inform further refinement and tailoring of parenting programs for military families to optimize intervention benefits. Our results also highlighted the distinctive roles of supportive vs. unsupportive PES, which appear to function differently to influence child adjustment. Identifying both mechanisms offers a more nuanced understanding of PES and its impact on child adjustment. Given the complexity of the model and demographic distinctions between fathers and mothers, we conducted separate models. Future research could consider triadic analyses to further strengthen understanding of the dynamic, interdependent nature of family processes.

## Data Availability

The data and code used in the current paper are available on request from the corresponding author.

## Declarations

### Clinical Registration

The research reports secondary data analysis from two pre-registered clinical trials: NCT02973906 and NCT03522610.
